# Avidity and Bystander Suppressive Capacity of Human Regulatory T Cells Expressing *De Novo* Autoreactive T-Cell Receptors in Type 1 Diabetes

**DOI:** 10.3389/fimmu.2017.01313

**Published:** 2017-10-26

**Authors:** Wen-I Yeh, Howard R. Seay, Brittney Newby, Amanda L. Posgai, Filipa Botelho Moniz, Aaron Michels, Clayton E. Mathews, Jeffrey A. Bluestone, Todd M. Brusko

**Affiliations:** ^1^Department of Pathology, Immunology, and Laboratory Medicine, University of Florida, Gainesville, FL, United States; ^2^Barbara Davis Center for Diabetes, University of Colorado School of Medicine, Aurora, CO, United States; ^3^Department of Microbiology and Immunology, University of California, San Francisco, San Francisco, CA, United States

**Keywords:** type 1 diabetes, regulatory T cells, T cell receptor, avidity, suppression mechanisms, adoptive cellular therapies, antigen-specific T cells, glutamic acid decarboxylase 65

## Abstract

The ability to alter antigen specificity by T-cell receptor (TCR) or chimeric antigen receptor (CAR) gene transfer has facilitated personalized cellular immune therapies in cancer. Inversely, this approach can be harnessed in autoimmune settings to attenuate inflammation by redirecting the specificity of regulatory T cells (Tregs). Herein, we demonstrate efficient protocols for lentiviral gene transfer of TCRs that recognize type 1 diabetes-related autoantigens with the goal of tissue-targeted induction of antigen-specific tolerance to halt β-cell destruction. We generated human Tregs expressing a high-affinity GAD_555–567_-reactive TCR (clone R164), as well as the lower affinity clone 4.13 specific for the same peptide. We demonstrated that *de novo* Treg avatars potently suppress antigen-specific and bystander responder T-cell (Tresp) proliferation *in vitro* in a process that requires Treg activation (*P* < 0.001 versus unactivated Tregs). When Tresp were also glutamic acid decarboxylase (GAD)-reactive, the high-affinity R164 Tregs exhibited increased suppression (*P* < 0.01) with lower Tresp-division index (*P* < 0.01) than the lower affinity 4.13 Tregs. These data demonstrate the feasibility of rapid expansion of antigen-specific Tregs for applications in attenuating β-cell autoimmunity and emphasize further opportunities for engineering cellular specificities, affinities, and phenotypes to tailor Treg activity in adoptive cell therapies for the treatment of type 1 diabetes.

## Introduction

T-cell receptor (TCR) transgenic regulatory T cells (Tregs) may represent a promising personalized treatment for T-cell-mediated autoimmune diseases such as type 1 diabetes. A curative therapy that targets the underlying immunological cause of disease to restore antigen-specific immunological tolerance represents an essential objective for the preservation of β-cell mass and function in the treatment of type 1 diabetes (1). Non-antigen-specific therapies involving hematopoietic stem cell transplantation combined with T-cell depletion, *via* high-dose anti-thymocyte globulin (ATG) or fludarabine, plus immunomodulation with cyclosporine and granulocyte-colony stimulating factor (G-CSF) have been shown to preserve β-cell function (2, 3), but the risks associated with these aggressive protocols preclude common clinical use. Comparatively, non-specific polyclonal immunotherapies, including immunoregulatory or depleting agents [e.g., alefacept (human LFA-3/IgG1-Fc fusion protein), teplizumab or otelixizumab (anti-CD3), and rituximab (anti-CD20)], have been better tolerated and offered some temporary efficacy but not long-term induction of tolerance (4–10). Until recently, most antigen-specific tolerance induction efforts have involved mucosal or peripheral administration of autoantigen(s), but thus far, such attempts have yielded limited efficacy in only a subset of patients, again with no indication for long-term tolerance induction (11, 12). Indeed, a safe treatment that controls persistent immune memory and induces long-term tolerance is needed.

Islet cell antigen-reactive Tregs, isolated from BDC2.5 TCR transgenic mice, could be expanded *in vitro*, and following adoptive transfer, migrate to the pancreatic draining lymph node/nodes (13). These Treg prevent and reverse autoimmune diabetes in non-obese diabetic (NOD) mice (14). In contrast, Tregs isolated and expanded from GAD286 TCR transgenic mice could suppress responder T cells (Tresp) *in vitro* but did not proliferate *in vivo* after transfer into recipient animals (14). Moreover, expression of cognate autoantigen is required for efficient trafficking of Tregs to the target organ and suppression of diabetes in NOD mice (15). These preclinical data support the notions that autoantigen-specific Tregs may offer an important therapy for type 1 diabetes, but also that intrinsic factors such as TCR specificity and/or avidity may play an important role in determining the capacity for immunomodulation and efficacy. The need for continued autoantigen expression by the host may render insulin-reactive TCRs less effective in patients with long-standing type 1 diabetes and support a need to investigate additional, potentially bystander, TCRs specific for additional/alternative autoantigen targets such as glutamic acid decarboxylase (GAD). Moreover, antigen localization, density, and persistence in β-cells along with risk of effector cell reprogramming support the use of alternative TCRs (16).

Genetically modified T cells with TCRs specific for tumor or viral antigens have become a valuable tool for the treatment of certain cancers or infections in humans (17–19). We previously demonstrated successful HLA class I-restricted TCR gene transfer in human Tregs using a high-affinity model receptor specific for the melanoma antigen tyrosinase presented by HLA-A*02:01 (20). We also generated a murine form of these tyrosinase-specific Tregs, and when transferred *in vivo*, the cells were capable of suppressing anti-tumor immunity in murine tumor models (20). This prompted us to ask whether candidate TCRs specific for type 1 diabetes-related autoantigens could be used to generate regulatory TCR avatars for human therapy.

Two TCR clones (R164 and 4.13) specific for the same β-cell peptide (GAD_555–567_) presented by HLA-DR4, but with different binding affinities, have been identified from the peripheral blood of subjects with or at-risk for T1D (21–23). Indeed, we recently identified T cells expressing the TCR β-chain complementarity determining region (CDR3β) of the GAD 4.13 clone from tissues of seven organ donors with type 1 diabetes, including the pancreatic islets of one type 1 diabetes subject. Interestingly, for one donor with long-standing disease, the TCR CDR3β was highly enriched in the pLN (>25% of all productive sequences), representing the most prevalent clone in both the Treg and conventional CD4^+^ T-cell (Tconv) populations (24). Interestingly, 4.13 TCR transgenic HLA-DR4 mice were reported to contain a mixture of Th1 and Tr1 cells capable of producing IL-10 (21). Conversely, R164 TCR transgenic HLA-DR4 mice exhibited greater thymic negative selection, and the T cells that escaped the thymus were skewed toward a Th1 phenotype (21). These observations support the notion that TCR avidity may impart important functional distinctions.

In a recent report by Ali et al., human CD4^+^ T cells were engineered to express the R164 TCR clone, and importantly, when administered to NSG-Ab^0^ DRB*04:01 mice, these R164 cells established long-term engraftment and islet infiltration, up to 12 weeks, without graft versus host disease (GvHD) (25). The creation of these autoreactive T-cell avatars presents the exciting possibility of autologous Treg therapy for type 1 diabetes with the benefit of antigen specificity to potentially enhance Treg trafficking to the target organ and associated draining lymph nodes. These antigen-specific Tregs would likely represent a significant improvement upon autologous polyclonal Treg therapy, which has already been shown to be safe for use in human subjects (26, 27). Indeed, antigen-specific Tregs offer the potential for long-term tolerance to the target antigen and possibly, to other key β-cell epitopes *via* bystander suppression and infectious tolerance (14, 28). To expand on these efforts, we generated primary human Tregs expressing the two GAD_555–567_-reactive TCR clones (R164 and 4.13), and investigated the pre-transfer conditions needed to optimize suppressive activity for potential use in adoptive cell therapy.

## Research Design and Methods

### Design and Synthesis of Lentiviral Constructs

**Figure 1:**
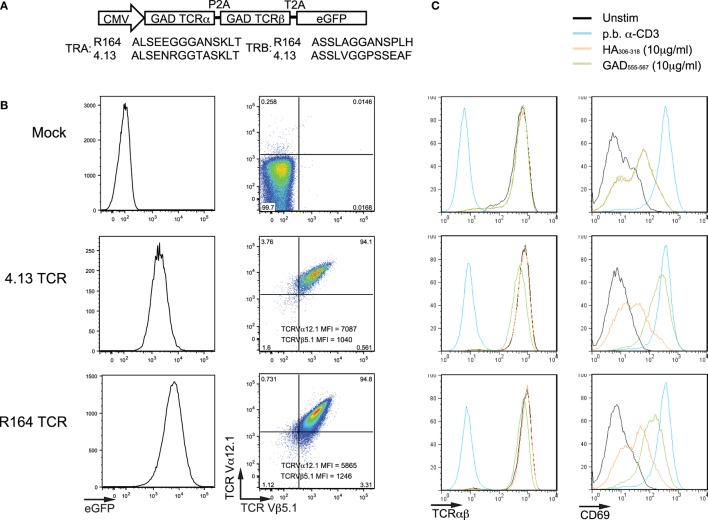
Verifying transfection and activation of Jurkat cells expressing T-cell receptor (TCR) clones. **(A)** Lentiviral constructs were designed containing the TCR α- and TCR β-chain genes (TRA and TRB, respectively) for known glutamic acid decarboxylase (GAD)-reactive clones (R164 and 4.13, additional clone information is listed in Table 1). The TRA and TRB coding regions were joined by a multicystronic P2A element, and TRB was linked by a multicystronic T2A element to an enhanced green fluorescent protein (eGFP) reporter. Amino-acid sequences for the complementarity determining region 3 (CDR3) are shown for both clones. **(B)** Jurkat T cells were untransduced (Mock; top), transduced with lentivirus expressing the R164 TCR (middle), and lentivirus expressing the 4.13 TCR (bottom), and expression was confirmed by flow cytometry. Double positivity for TCR Va12.1 and Vβ5.1, which is comparable between both clones, and eGFP indicates successful transduction. Untransduced cells were negative for both markers. **(C)** Mock (top), 4.13 (middle), and R164 (bottom) TCR-transduced cells were unstimulated (black), stimulated with plate bound anti-CD3 (p.b. α-CD3, blue), irrelevant antigen influenza hemagglutinin (HA_306–318_, orange), or cognate antigen (GAD_555–567_, green). TCR expression (left panels) was comparable across all unstimulated, HA-stimulated, and GAD-stimulated cells, and p.b. α-CD3 stimulation induced TCR downregulation. p.b. α-CD3 stimulation also induced the highest level of CD69 expression (right panels), and unstimulated cells exhibited low CD69 expression. These observations were comparable across mock, R164, or 4.13 transduced cells.

Lentiviral vectors were generated to express TCR clones 4.13 and R164, both of which react to GAD_555–567_ (21, 25) (Table 1). Equimolar expression of TCR α- and β-chains was achieved by inclusion of a multicystronic P2A element, followed by a T2A element and the reporter, enhanced green fluorescent protein (eGFP). The constructs were cloned into pCNFW lentiviral vectors with expression driven by a cytomegalovirus promoter as previously described (25) (Figure 1A). Lentiviral vectors containing the Melan-A reactive TCR clone melanoma antigen recognized by T cells 1 (MART-1) were generated as previously described (29) (Table 1).

**Table 1:** T-cell receptor (TCR) clone information.

TCR (IMGT)		TRA gene			TRB gene				pMHC restriction		Source
S. no.	Clone	V	J	CDR3 AA sequence	V	D	J	CDR3 AA sequence	HLA	Antigen	
1	5	TRAV21	TRAJ6	CAVKRTGGSYIPTF	TRBV11-2	TRBD1	TRBJ2-2	CASSSFWGSDTGELFF	DQ8	InsB (9–23)	Roep, personal communication
2	GSE.20D11^a^	TRAV12-3	TRAJ4	CAILSGGYNKLIF	TRBV02-01*01	TRBD02-01	TRBJ02-05*01	CASSAETQYF	DQ8	InsB (9–23)	(30)
3	GSE.6H9^a^	TRAV26-1	TRAJ40	CIVRVDSGTYKYIF	TRBV7-2	TRBD2	TRBJ2-1	CASSLTAGLASTYNEQFF	DQ8/DQ8-trans	InsB (9–23)	(30)
4	T1D#3 C8	TRAV17	TRAJ23	CATDAGYNQGGKLIF	TRBV5-1	TRBD2	TRBJ1-3	CASSAGNTIYF	DQ8	InsB (9–23)	(31)
5	T1D#10 C8	TRAV12-3	TRAJ26	CATAYGQNFVF	TRBV4-1	TRBD2	TRBJ2-2	CASSRGGGNTGELFF	DQ8	InsB (9–23)	(31)
6	PM1#11	TRAV35*02	TRAJ54*01	CAGHSIIQGAQKLVF	TRBV5-1*01	TRBD2*02	TRBJ2-1*01	CASGRSSYNEQFF	DRB1*03:01	GAD (339–352)	(32)
7	MHB10.3	TRAV4*01	TRAJ27*01	CLVGDSLNTNAGKSTF	TRBV29-1*01	TRBD2*01	TRBJ2-2*01	CSVEDRNTGELFF	DRB1*03:01	InsB (11–30)	(33)
8	SD32.5	TRAV26-1*01	TRAJ23*01	CIVRVSSAYYNQGGKLIF	TRBV27*01	TRBD2*01	TRBJ2-3*01	CASSPRANTDTQYF	DRB1*04:01	InsA (5–21)	(34)
9	SD52.c1	TRAV4*01	TRAJ27*01	CLVGDSLNTNAGKSTF	TRBV27*01	TRBD1*01	TRBJ1-5*01	CASSWSSIGNQPQHF	DRB1*04:01	PPI (C18–A1)	(34)
10	R164	TRAV19*01	TRAJ56*01	CALSEEGGGANSKLTF	TRBV05-01*01	TRBD02-01*01	TRBJ01-06*01	CASSLAGGANSPLHF	DRB1*04:01	GAD (555–567)	(23)
11	4.13	TRAV19*01	TRAJ44*01	CALSENRGGTASKLTF	TRBV05-01*01	TRBD01-01*01	TRBJ01-01*01	CASSLVGGPSSEAFF	DRB1*04:01	GAD (555–567)	(21)
12	1E6	TRAV12-3	TRAJ12	CAMRGDSSYKLIF	TRBV12-4	TRBD2	TRBJ2-4	CASSLWEKLAKNIQYF	A*02-01	PPI (15–24)	(35)
13	D222D	TRAV17*01	TRAJ36*01	CAVTGANNLFF	TRBV19*01	TRBD1*01	TRBJ2-2*01	CASSIEGPTGELFF	A*02-01	ZnT8 (186–194)	Patent WO2017046335 A1
14	32	TRAV12-1	TRAJ48*01	CVVNILSNFGNEKLTF	TRBV20	TRBD01-01*01	TRBJ2-01*01	CSASRQGWVNEQFF	A*02-01	IGRP (265–273)	(36)
15	MART-1	TRAV12-2	TRAJ23	CAVNFGGGKLIF	TRBV6-4	TRBD2	TRBJ1-1	CASSLSFGTEAFF	A*02-01	Melan-A (27–35)	(37)

*For the experiments described herein, T cells were transduced to express TCR clones 4.13 or R164, which were first identified from the peripheral blood or pancreas of a type 1 diabetes patient or an autoantibody positive subject at risk for T1D. CD8^+^ T cells were transduced to express melanoma antigen recognized by T cells 1 (MART-1) TCR. Remaining TCR clones [sourced from the international ImMunoGeneTics information system^®^, IMGT.org (IMGT)] listed are those with known reactivities to type 1 diabetes-related autoantigen peptides with which we can generate lentivirus constructs to create additional Treg avatars. TCRα (TRA) and TCRβ (TRB) V, D, and J genes as well as complementarity determining region 3 (CDR3) amino acid (AA) sequence, HLA restriction, and antigen target are listed for each clone*.

*^a^Intra-islet source material*.

### Lentivirus Production

Lentiviral vectors were generated as described (20). Briefly, 55 µg of lentiviral vector and 18.3 µg of each helper plasmid were co-transfected in 293T cells using TransIT-2020 transfection reagent (Mirus, Madison, WI, USA). Supernatants were collected 72 h after transfection, filtered through a 0.45-µm filter, and concentrated by ultracentrifugation at 198,000 × *g* for 1.5 h.

### Subject Enrollment and T-Cell Isolation

Healthy control blood donors provided written informed consent prior to inclusion in the study in accordance with the Declaration of Helsinki and according to Institutional Review Board-approved protocols at the University of Florida (Protocol no. IRB201600092) and the University of Colorado Denver (Protocol no. COMIRB92-292). T cells where enriched by negative selection from whole blood by Ficoll-Paque density gradient in combination with a total T-cell enrichment cocktail by following manufacturer’s instructions (Catalog no. 15061, STEMCELL Technologies, Cambridge, MA, USA). Cells were stained with fluorescently labeled antibodies [CD4-PB (clone RPA-T4), CD8-APC.H7 (SK1), CD25-APC (BC96), CD127-PE (A019D5), and CD45RA-PE-Cy7 (HI100)]. CD4^+^CD25^+^CD127^lo/−^ Tregs, CD4^+^CD25^−^CD127^+^CD45RA^+^ naïve Tconv cells, and CD8^+^CD45RA^+^ naïve CD8^+^ T cells were purified by fluorescence-activated cell sorting (FACS) using a BD FACSAria III (BD Biosciences, San Jose, CA, USA).

### Lentiviral Transduction (LV TD) of Human T Cells

#### Jurkat Cells

Human Jurkat T cells were plated at 2 × 10^5^ cells/well in a 24-well plate and transduced in the presence of protamine sulfate (8 µg/mL; Sigma-Aldrich, St. Louis, MO, USA). Transgene expression was assessed 72 h post-transduction by flow cytometry (Figure 1).

#### Primary Human T Cells

**Figure 2:**
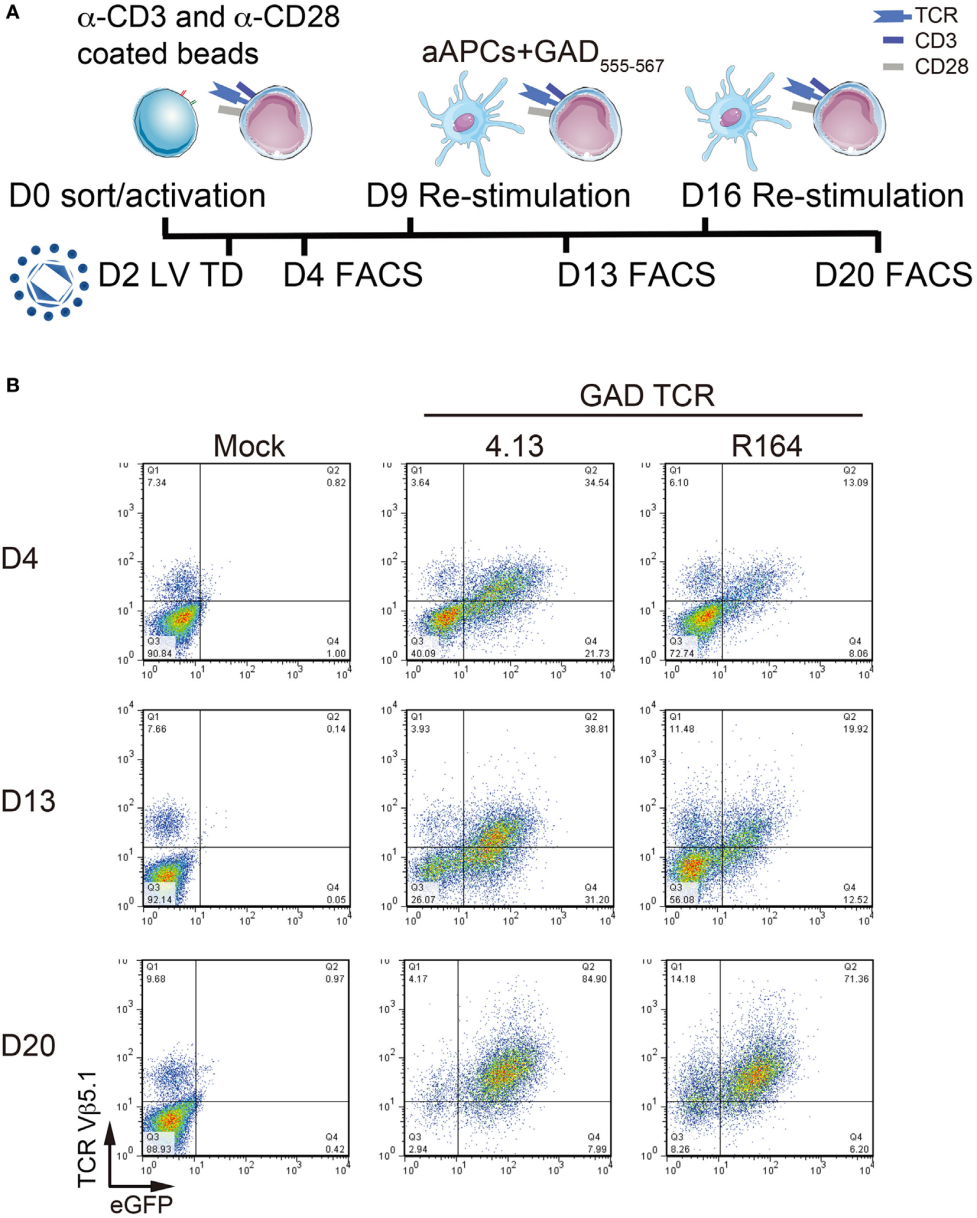
Serial activation increases transduction efficiency. **(A)** Primary CD4^+^ T cells remain untransduced (Mock) or transduced with lentivirus (LV TD) expressing T-cell receptor (TCR) clones 4.13 or R164 were activated with α-CD3/α-CD28 coated beads on day 0 (D0). Cells were restimulated with artificial APC (aAPCs; K562 cell line expressing HLA-DR4) and GAD_555–567_ peptide for an additional two rounds on day 9 (D9) and day 16 (D16). IL-2 (30 IU/mL) was given every 2–3 days. Transduction efficiency was detected by fluorescence-activated cell sorting (FACS) every 4 days after stimulation (D4, D13, and D20). **(B)** TCR Vβ5.1 and enhanced green fluorescent protein (eGFP) reporter were assessed by flow cytometry on day 4 (D4, top), day 13 (D13, middle), and day 20 (D20, bottom). At each time point, a portion of untransduced cells were positive for TCR Vβ5.1, but no eGFP was observed. TCR Vβ5.1 and eGFP positivity was observed for 4.13 and R164 transduced cells at each time point, and the proportion of dual positive cells increased with time.

**Figure 3:**
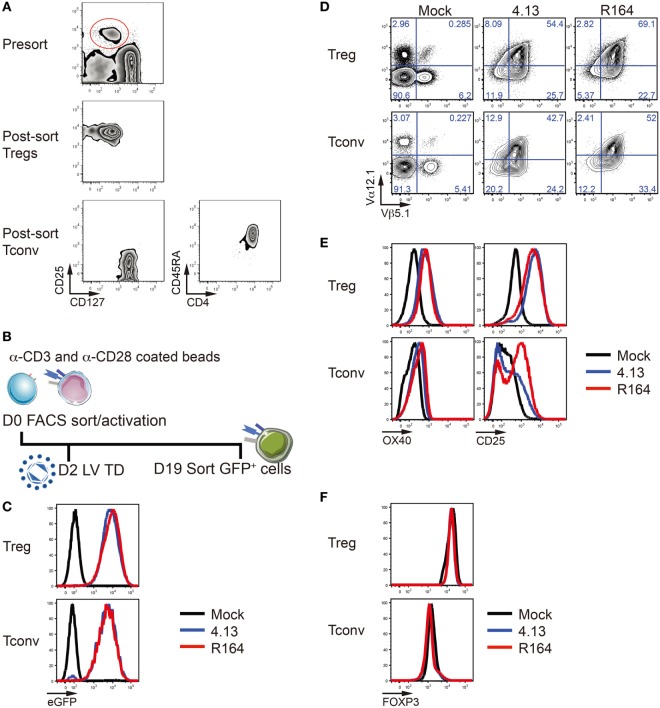
Verification of T-cell receptor (TCR) overexpression and stability of transfected regulatory T cells (Tregs). **(A)** CD25^+^CD127^lo/−^ Tregs (top and middle) and CD25^lo/−^CD127^+^CD4^+^CD45RA^+^ naïve conventional T cells (Tconv, bottom) were purified from adult peripheral blood by fluorescent-activated cell sorting (FACS). **(B)** Tregs were activated with α-CD3/α-CD28 coated beads on day 0 (D0). Lentiviral transduction (LV TD) was performed on day 2 (D2). Successfully transduced cells expressing enhanced green fluorescent protein (eGFP) were FACS-purified on day 19 (D19) for further analysis. **(C)** Treg (top) and Tconv cells (bottom) were untransduced (Mock; black), transduced with lentivirus containing the expression vector for the GAD-reactive TCR clone 4.13 (blue) or R164 (red), and eGFP expression among TCR transduced cells was confirmed by flow cytometry. **(D)** Mock (left), 4.13 (middle), and R164 (right) transduced Tregs (top) and Tconv cells (bottom) were stained for the TCRVα12.1 and TCRVβ5.1 chains that comprise the TCR clones 4.13 and R164, and overexpression was confirmed for TCR transduced cells. **(E)** Cell activation markers OX40 and CD25 were measured after co-culturing T cells with HLA-DR4 expressing K562 artificial antigen-presenting cells (aAPCs) loaded with GAD GAD_555–567_ peptide for 1 day. **(F)** The majority of Tregs in all conditions (non-transduced, 4.13, or R164) maintain FOXP3 expression (top left). Low frequency of FOXP3 expression was observed in Tconv (bottom left).

Primary human T cells were transduced as previously described (3). Briefly, FACS-purified CD4^+^ T cells (total), Tregs, naïve Tconv cells, and naïve CD8^+^ T cells were plated at 2.5 × 10^5^ cells/well in a 24-well plate. Total CD4^+^ T cells, naïve Tconv, and CD8^+^ T cells were activated with anti-CD3 and anti-CD28 dynabeads (Catalog no. 11161D, ThermoFisher Scientific, Waltham, MA, USA), while Tregs were expanded with anti-CD3 and anti-CD28 conjugated microbeads (Catalog no. 130-091-441, Miltenyi Biotec, San Diego, CA, USA) according to the manufacturer’s instructions. After 48 h of activation, cells were supplied with protamine sulfate (8 µg/mL) and transduced with 3 TU/cell of lentivirus for TCR expression followed by spinoculation. Total CD4^+^ T cells were supplied IL-2 (30 IU/mL) every 2–3 days and restimulated with the HLA-DR4 (DRB1*04:01) expressing K562 artificial antigen-presenting cell (aAPC) line and GAD_555–567_ peptide on day 9 and day 16 for serial activation (Figure 2). For T-cell subsets, IL-2 (300 IU/mL for Treg; 20 IU/mL for Tconv; 100 IU/mL for CD8^+^ T cells) was supplied every 1–2 days during expansion (Figure 3). K562 aAPCs were kindly provided by Drs. James Riley and Bruce Levine (University of Pennsylvania).

### Flow Cytometry

Cells were first stained with live/dead near-IR (Invitrogen) followed by fluorescently labeled antibodies specific for the following surface markers: CD4-PB (clone RPA-T4), CD69-BV711 (FN50), TCR Vα12.1-Alexa Fluor 647 (6D6.6), TCR Vβ5.1-PE (IMMU 157), OX40-APC (ACT35), and CD25-PE (BC96). The TCR Vα12.1 monoclonal antibody was labeled with Alexa Fluor 647 using Zenon labeling kit (ThermoFisher Scientific, Waltham, MA, USA) before staining. Intracellular FOXP3 was stained using a FOXP3-Alexa Fluor 488 (206D) antibody with a FOXP3/transcription factor staining kit (Catalog no. 00-5523-00, ThermoFisher Scientific, Waltham, MA, USA) according to the manufacturer’s instructions. Flow cytometry data were collected using an LSRFortessa (BD Biosciences) and analyzed with FlowJo software (Tree Star, Ashland, OR, USA).

### Treg Suppression Assay

T-cell-receptor-redirected Tregs were FACS-purified based on their eGFP expression and tested for the ability to suppress polyclonal or TCR-transduced Tresp proliferation, as described previously (38). For suppression assays involving polyclonal Tresp, cells were stimulated with 2 µg/mL soluble anti-CD3 (clone Hit3a) and 1 µg/mL soluble anti-CD28 (clone 28.2, BD PharMingen). Proliferation was determined by the incorporation of ^3^H-thymidine by pulsing cultures with 1 mCi of ^3^H-thymidine for the final 12–16 h of culture. Plates were harvested on a Packard FilterMate harvester and read on a Packard TopCount Scintillation & Luminescence Counter (Perkin Elmer; Waltham, MA, USA). Interferon-gamma (IFN-γ) was measured from the supernatant by ELISA. For suppression assays involving TCR-redirected Tresp, Tregs expressing the 4.13 TCR were stained with cell proliferation dye eFluor670 (5 µM; Catalog no. 65-0840-85, ThermoFisher Scientific, Waltham, MA, USA), whereas Tresp expressing the 4.13 TCR or a Melan-A_27–35_ reactive MART-1 TCR were labeled with CellTrace Violet (5 µM; Catalog no. C34571, ThermoFisher Scientific, Waltham, MA, USA) following the manufacturer’s instructions. Tregs were plated in two-fold serial dilution, co-cultured with Tresp, and activated with the indicated peptide presented by irradiated CD3-depleted peripheral blood mononuclear cells (PBMCs) (HLA-DRB1*04:01 and A*02:01) for 3–4 days. Triplicate cultures were pooled, harvested, stained with live/dead dye and for the surface markers, CD4 and CD8, and then analyzed by flow cytometry as described above. Proliferation was calculated by division and replication index of Tresp cells. Assay conditions are detailed in Table S1 in Supplementary Material.

### Statistical Analysis

Data were analyzed by two-way analysis of variance (ANOVA) and graphs prepared using GraphPad Prism version 6 software (La Jolla, CA, USA).

## Results

### Validation of TCR Expression and Activation in Human Jurkat Cells

Two lentiviral constructs with identical backbone each contained the TCR α- and TCR β-chain genes (TRA and TRB, respectively) for the GAD_555–567_-reactive clones R164 or 4.13 followed by an eGFP reporter sequence as shown in Figure 1A. Multi-cystronic and equal molar expression of TCR α- and β-chains is achieved by including P2A and T2A elements between TRA, TRB, and the eGFP reporter. We used lentivirus carrying these constructs to transduce human Jurkat cells and express one of the two *de novo* TCRs. As expected, untransduced cells did not express eGFP, TCR α-chain V gene family 12.1 (TCRVα12.1), and TCR β-chain V gene family 5.1 (TCRVβ5.1), which are common to both R164 and 4.13 clones (Figure 1B). Over 94% of Jurkat cells transduced with either the R164 or 4.13 TCR lentiviral construct were double positive for both TCRVα12.1 and Vβ5.1 with comparable mean fluorescence intensity (MFI) (Figure 1B). To verify stable transfection and antigen-specific activation of Jurkat cell lines, we stimulated mock (eGFP^−^), R164, and 4.13 transduced cells with K562 aAPCs loaded with cognate antigen (GAD_555–567_) and evaluated for TCR and CD69 expression levels. Positive and negative controls included stimulation of transduced cells with plate bound anti-CD3 or K562 aAPCs loaded with “irrelevant” antigen influenza hemagglutinin (HA_306–318_), respectively. Compared with unstimulated cells, anti-CD3 induced TCR downregulation concurrent with high expression of the activation marker CD69 in each of the three cell lines (Figure 1C). GAD_555–567_ stimulation of both R164 and 4.13 cell lines resulted in high CD69 expression without TCR downregulation, whereas irrelevant antigen resulted in only modest upregulation of CD69, likely due to interaction with the costimulatory molecule, CD80 constituently expressed by the aAPCs (Figure 1C). These data support both surface receptor expression and activation in the presence of the cognate peptide presented by HLA-DR4.

### Optimizing TCR Expression in Primary Human CD4^+^ T Cells

We next transduced primary human peripheral blood CD4^+^ T cells to express the GAD-reactive 4.13 and R164 TCRs and assessed transduction efficiency. Cells were stimulated with anti-CD3/anti-CD28 coated beads on day 0, transduced on day 2, and restimulated on days 9 and 16 with K562-DR4 aAPCs loaded with GAD_555–567_ peptide (Figure 2A). As expected, a portion of untransduced cells expressed TCRVβ5.1 but not eGFP (Figure 2B). In addition, 35% of GAD 4.13 and 13% of GAD R164 cells were TCRVβ5.1^+^eGFP^+^ on day 4, and by day 20, 85 and 71% of 4.13 and R164 cells, respectively, were double positive for TCRVβ5.1 and eGFP (Figure 2B) suggesting that serial activation resulted in enriched T-cell avatars.

### TCR Expression in Primary Human Regulatory and Conventional T-Cell Subsets

CD4^+^CD25^+^CD127^lo/−^ Tregs and CD4^+^CD25^−^CD127^+^CD45RA^+^ naïve Tconv were FACS-purified from peripheral blood (Figure 3A). We then generated primary human Tregs and Tconv expressing the GAD 4.13 and GAD R164 TCRs and expanded them with anti-CD3/CD28-coated beads for 19 days (Figure 3B). Again, compared with untransduced cells, 4.13 and R164 cells were confirmed to express high levels of eGFP (Figure 3C) as well as the Vα12.1 and Vβ5.1 chains of the GAD-reactive TCRs as measured by flow cytometry (Figure 3D). The activation markers OX40 and CD25 were upregulated on 4.13 and R164 transduced Tregs compared with mock transduced Tregs 1 day post co-culture with HLA-DRB1*04:01 expressing K562 aAPC loaded with cognate peptide (Figure 3E). Similarly, OX40 was slightly upregulated on the surface of 4.13 and R164 transduced Tconv following aAPC-antigen activation, while CD25 upregulation was more pronounced for R164 Tconv compared with 4.13 Tconv (Figure 3E) (39). After transduction and anti-CD3/28 stimulation, Tregs maintained FOXP3 positivity whereas Tconv cells showed low/intermediate expression of FOXP3 (Figure 3F) indicating transduction affected neither Treg differentiation nor development.

### Suppressive Capacity of R164 and 4.13 Treg Avatars

**Figure 4:**
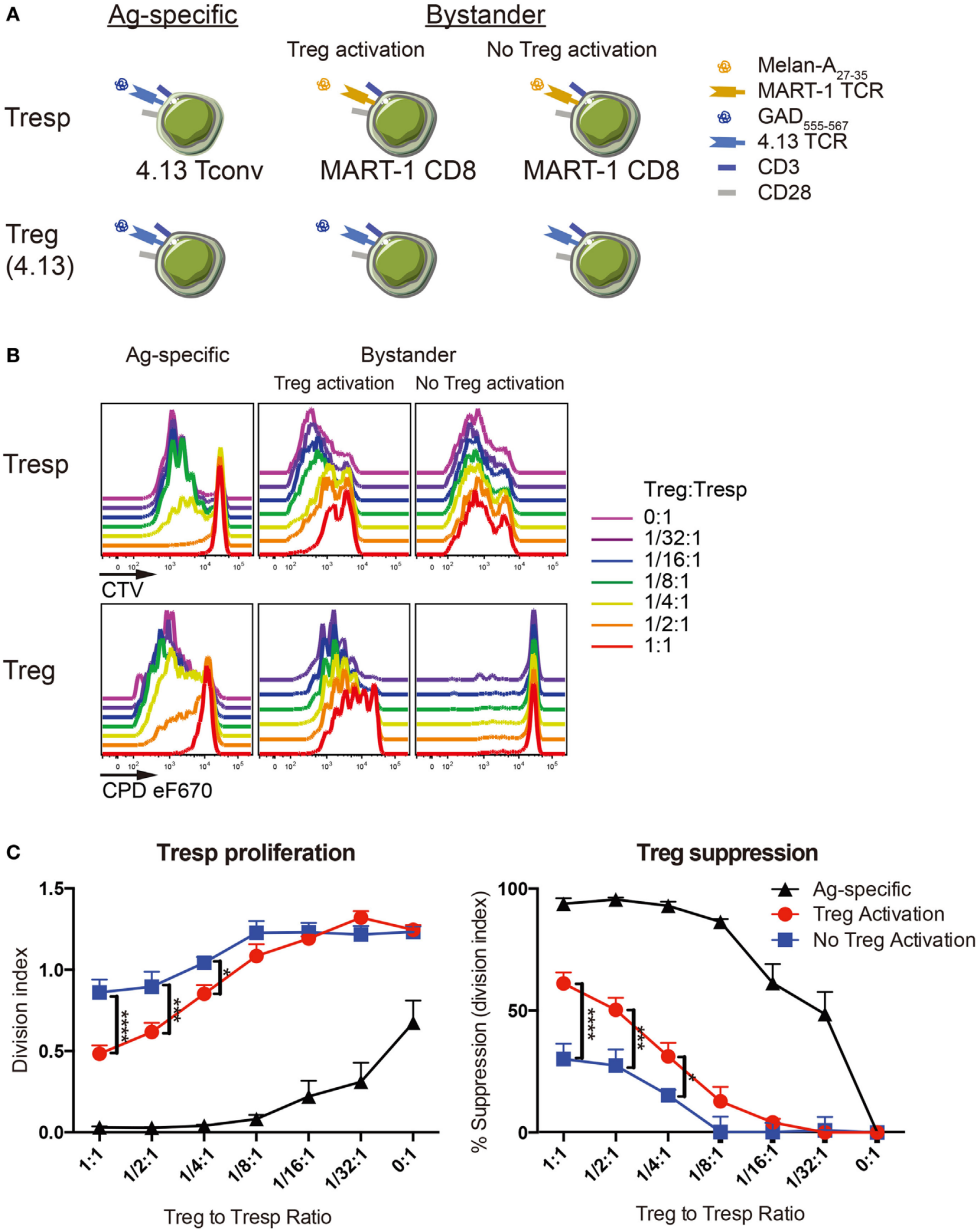
Regulatory T-cell (Treg) suppression is optimal with activation. **(A)** Antigen-specific suppression by 4.13 Tregs was tested on 4.13 T-cell receptor (TCR) transduced conventional T cells (Tconv) *in vitro* (left). Bystander suppression by 4.13 Tregs was assessed on CD8^+^ T cells expressing the melanoma antigen recognized by T cells 1 (MART-1) TCR, with (middle) or without (right) Treg activation. **(B)** Tregs were isolated from adult peripheral blood and transduced to express glutamic acid decarboxylase (GAD) 4.13 TCR. Transduced Tregs were sorted, labeled with cell proliferation dye (CPD) eFluor670, and plated in decreasing proportions with GAD 4.13 TCR transduced CD4^+^ responder T cells (Tresp) (Ag-specific) or MART-1 transduced CD8^+^ Tresp (Bystander) stained with cell trace violet (CTV) dye. For Ag-specific suppression, GAD 4.13 Tresp and Treg were activated with cognate GAD_555–567_ peptide presented by CD3-depleted peripheral blood mononuclear cell (PBMC) from an HLA-DR4 individual. For bystander suppression, MART-1 CD8^+^ Tresp and GAD 4.13 Tregs were activated with Melan-A_27–35_ with or without GAD_555–567_ peptide, again presented by CD3-depleted PBMC from an HLA-DR4 individual. Cell proliferation was evaluated *via* dye dilution for Tresp (top) and Tregs (bottom). Tresp proliferation decreased as the Treg to Tresp ratio increased only when Tregs were activated, and suppression was most effective when Treg activation was antigen-specific. Unactivated Tregs exhibited little to no proliferation. **(C)** Suppression was evaluated by Tresp division index (left) and percent (%) suppression (right). Tresp division index was significantly lower and percent suppression of Tresp proliferation was significantly greater in antigen-specific settings (Ag-specific, black) followed by bystander suppression when Tregs were activated (red). Two-way analysis of variance (ANOVA) (*P < 0.05, ***P < 0.001, ****P < 0.0001).

The capacity to impact type 1 diabetes progression prior to symptomatic onset (i.e., in the context of multiple autoantibody positive high-risk individuals) or at the time of symptomatic disease will likely require the capacity to control a polyclonal memory T-cell response. Depletion of these cells is one potential approach but would require broad targeting resulting in a period of immunosuppression. We hypothesize that tissue targeting and dominant suppression of a broad repertoire by TCR-redirected Tregs may confer persistent tolerance. Therefore, we sought to understand if Treg avatars functionally suppress Tresp in an antigen-specific and/or bystander manner. We first demonstrated that LV TD does not affect Treg capacity to suppress polyclonal Tresp using well-described *in vitro* suppression assays (38). Both proliferation and IFN-γ production by polyclonal stimulated Tresp were comparable between R164, 4.13, and mock transduced Treg groups (Figure S1 in Supplementary Material). Then, we assessed Treg suppressive capacity in both antigen-specific and bystander mechanisms with or without Treg activation (Figure 4A). At physiological ratios, Tregs showed excellent suppression of Tresp against cognate antigen by culturing both CD4^+^ Tresp and Tregs engineered to express a GAD-reactive TCR clone 4.13 and activated with cognate GAD_555–567_ peptide (Figures 4B,C, Ag-specific; Table S1 in Supplementary Material). Specifically, Tresp division was significantly blunted, and Treg percent suppression was significantly greater than in settings of bystander suppression (Figure 4C).

Importantly, CD8^+^ T cells are thought to drive type 1 diabetes pathogenesis *in vivo* through the direct killing of β-cells (40). We therefore sought to understand whether Treg avatars are capable of suppressing CD8^+^ T cells in a bystander manner in the islets or periphery. We tested the capacity of GAD-specific Tregs to suppress MART-1 CD8^+^ T cells recognizing the tumor antigen Melan-A, with or without Treg activation. While unactivated 4.13 Tregs exhibited limited suppression of MART-1 CD8^+^ Tresp proliferation, GAD-activation of 4.13 Tregs resulted in significantly reduced Tresp proliferation and increased suppression of MART-1 CD8^+^ Tresp (Figures 4B,C). This supports two notions: first, that Treg activation is required for functional suppression and second, that TCR transgenic Treg avatars are capable of both antigen-specific and bystander suppression.

**Figure 5:**
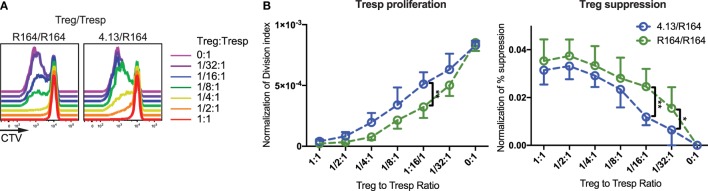
High-avidity T-cell receptor (TCR) activation augments regulatory T-cell (Treg) suppression. **(A)** Tregs expressing the high-avidity GAD_555–567_-reactive R164 TCR (left) or the low-avidity GAD_555–567_-reactive 4.13 TCR (right) were activated with their cognate antigen. Activated Tregs were plated in decreasing proportions with Tresp expressing the R164 TCR and stained with cell trace violet (CTV) dye as indicated in the figure. Tresp proliferation was evaluated *via* dye dilution. Both Treg clones were able to suppress Tresp proliferation at 1:1 cell ratio, but R164 Tregs were more effective in suppressing Tresp proliferation at lower ratios (1/2:1–1/16:1). **(B)** Both Tresp proliferation and Treg suppression were normalized to the reporter enhanced green fluorescent protein (eGFP) mean fluorescence intensity (MFI) to control for potential variation in TCR expression levels. The Tresp division index (left) was significantly lower and percent (%) suppression (right) based on replication index was significantly greater for suppression assays using high-avidity R164 Tregs (green) compared with 4.13 Tregs (blue).

Finally, we examined if TCR avidity affects Treg suppressive ability with the advantage of using two GAD_555–567_-reactive TCR clones, R164 and 4.13, where R164 exhibits higher avidity. Either GAD R164 or 4.13 Tregs were cultured with R164 CD4^+^ Tresp in the presence of peptide presented by CD3-depleted APCs from HLA-DRB1*04:01/A*02:01 individuals. We normalized the Treg suppression capacity against reporter eGFP MFI allowing us control for potential variation in TCR expression levels. Indeed, cells expressing the high-avidity R164 TCR were significantly more suppressive than cells expressing the lower avidity 4.13 TCR (Figure 5). These data support the notion that Treg avatars engineered to receive a higher affinity signal through the TCR are more efficient suppressors of bystander T-cell responses. It remains to be investigated how costimulatory signals will impact suppressive activity, a notion that may be particularly important for assessing signaling through CAR-T vectors if expressed by Tregs.

## Discussion

For tolerogenic adoptive cell therapy, autologous polyclonal Tregs can be expanded from peripheral blood which provides an attractive Treg source given the abundant cell numbers, allowing for repeat dosing if needed, and the safety associated with autologous cell therapy (26, 27). Concerns remain, however, regarding the lack of antigen specificity by administrating polyclonal Tregs. Indeed, Tregs have a highly diverse repertoire (24), which indicates the precursor frequency of autoreactive Tregs will likely be quite low in peripheral blood, especially considering that Tregs do not enrich to the extent that is observed for Tconv during conversion to effector T cells and expansion. Hence, we expect polyclonal Treg therapy to confer potentially limited efficacy and trafficking to the pancreas or PLN to induce immunological tolerance for β-cell antigens. To address this, we utilized LV TD to generate primary human T-cell avatars expressing two GAD_555–567_-reactive TCRs (R164 and 4.13) originally identified from the peripheral blood of subjects with or at risk for type 1 diabetes (21–23). These clones, which differ by only 10 amino acids in TRAV and TRBV genes and only three amino acid charge differences in the CDR3 region (Table 1), exhibit different binding affinities for their cognate antigen peptide (21).

Regulatory T cell avatars maintained FOXP3 positivity, indicating that LV TD did not impair Treg stability. Functionally, 4.13 Treg avatars effectively suppressed antigen-specific 4.13 CD4^+^ Tresp. Beyond this, 4.13 Treg avatars exhibited a moderate ability to suppress MART-1 CD8^+^ Tresp in a bystander suppressive mechanism that required Treg activation. Interestingly, when the high-avidity R164 or lower avidity 4.13 Treg avatars were cultured with R164 CD4^+^ Tresp in the presence of GAD_555–567_ peptide presented by CD3-depleted HLA-DRB1*04:01/HLA-A*02:01 APCs, R164 TCR were significantly more suppressive. This suggests that Treg TCR avidity affects suppressive ability and importantly, that the optimal avidity of TCR or CAR signals may be required for effective Treg avatar cellular therapy. Importantly, however, there is the potential for heterologous TCR chain pairing with the endogenous receptors, and further experiments are needed to empirically determine this for each receptor. Recent developments in gene editing technologies could be used to correct for this potential caveat. Specifically, knockout of endogenous TCR α- and β-chains *via* CRISPR/Cas9, silencing of endogenous TCR *via* shRNA with expression of a codon optimized *de novo* TCR, or the domain-swap approach described by Bethune et al. (41) could be applied.

Although preproinsulin (PPI) and alternative forms of this antigen (e.g., hybrid insulin peptides, alternative mRNA transcripts) (42, 43) are considered key type 1 diabetes autoantigens, we anticipate continued expression of cognate antigen will be imperative for Treg survival and trafficking to the target organ (15, 44). Hence, we focused our efforts on the development of Tregs against GAD65, which exhibits a high autoantigen density in T1D (45) and is the target of persistent autoimmunity, as evidenced by maintenance of autoantibodies (46). We anticipate that adoptive cell therapy with GAD-specific Tregs will lead to bystander suppression and infectious tolerance (47) with the hope for inducing long-term antigen-specific tolerance to GAD as well as other β-cell antigens. A recent report by Hull et al. described the generation and *in vitro* characterization of peripheral blood-derived human Tregs expressing TCRs specific for insulinoma-associated protein-2 (IA-2) and insulin (48). Although the authors did not conduct functional comparisons of TCR avidity, *in vivo* investigations of these and the GAD-specific clones generated herein are certainly warranted to determine the optimal clone(s) for tolerogenic cell therapy as we move forward toward clinical testing.

Chimeric antigen receptor (CAR) Treg therapy should also be considered given the promising outcomes observed from CAR effector T cells in cancer immunotherapy (49, 50). CAR Treg therapy could be particularly advantageous given that CAR T cells are not constrained by HLA restriction, hence, offering the opportunity for off-the-shelf clinical utility. However, the need for surface expression of the target antigen on islets or β-cells represents a clear limitation compared with TCR gene transfer, which allows for recognition of intracellular antigens in the context of class II HLA. An additional approach to potentially address this challenge could involve the use of a CD8-restricted TCR that functions independently of the CD8 co-receptor. In fact, this type of activity has been demonstrated previously with a high-affinity melanoma antigen tyrosinase-reactive TCR expressed by CD4^+^ T cells (20). Yet an additional approach could involve the identification of a CAR capable of recognizing an islet epitope in the context of HLA-A2, given the observation that beta cells hyperexpress class I HLA in settings of type 1 diabetes (51).

RNA TCR or CAR gene transfer has been demonstrated as one potential approach to confer T-cell antigen-specificity (52), and could be further explored in the context of tolerogenic adoptive Treg therapy for type 1 diabetes. Specifically, mRNA encoding the TCR or a CAR of choice can be introduced to T cells *via* electroporation, thereby eliminating the need for LV TD and associated safety requirements. This approach would, of course, be transient with transgene expression lasting only a few days (53), but might be accomplished with multiple autologous dosings. Temporary transgene expression presents lower risk of off-target effects such as bystander suppression of anti-tumor or anti-infection immunity. However, lentivirus transduced Treg avatars likely offer greater potential for long-term efficacy in clearing islet infiltration/inflammation and leading to persistent engraftment.

Importantly, adoptive cell therapy with polyclonal autologous peripheral blood Tregs has been demonstrated to be safe in Phase I clinical trials (26, 27). While we anticipate a similar safety profile with TCR transgenic Treg therapy, tolerogenic cell therapies always carry with them possible associations with increased risk of infection or cancer due to bystander suppression and infectious tolerance mechanisms. Thus, there is a need to perform Phase I safety studies and simultaneously, investigate co-transfection of suicide genes for inducible apoptosis of TCR transgenic Tregs—a biological “off-switch” (54).

We recently demonstrated that cryopreserved umbilical cord blood Tregs (cryoCB Tregs) could be isolated and expanded efficiently while retaining their naïve phenotype as well as suppressive capacity (55). Beyond the possibility for polyclonal autologous cryoCB Treg therapy, these cells offer the potential to generate antigen-specific Treg avatars from precursors with an optimal naïve phenotype and without the need for a large-volume peripheral blood draw and leukapheresis, which is generally contraindicated in pediatric patients. This is a goal currently being pursued by our lab and others. Additional optimization, such as further genetic manipulation of TCR transgenic Tregs, could be implemented to correct intrinsic single-nucleotide polymorphisms (SNPs) with putative implications for Treg function and known associations with type 1 diabetes as identified by genome-wide association studies (GWAS) (56). Beyond this, there is potential for delivery of tissue repair factors directly to the pancreas *via* production by antigen-specific Tregs or *via* conjugation to the Treg surface using poly lactic-co-glycolic acid (PLGA) nanoparticles (57–59). For these approaches to be successful, we expect that Treg survival *in vivo* and trafficking to the target organ will depend largely upon TCR specificity. Hence, we anticipate the functional effects of TCR avidity on human Treg phenotype and function, as demonstrated herein, will be extremely important as we refine adoptive cell therapies to reverse autoimmunity in type 1 diabetes.

## Ethics Statement

Healthy control blood donors provided written informed consent prior to inclusion in the study in accordance with the Declaration of Helsinki and according to Institutional Review Board-approved protocols at the University of Florida (Protocol no. IRB201500059) and the University of Colorado Denver (Protocol no. COMIRB92-292).

## Guarantor Statement

As the guarantor of this work, Todd Brusko assumes responsibility for ethical completion of the study, integrity of the data, and accuracy of the data analysis reported herein.

## Author Contributions

W-IY researched and analyzed the data and wrote the manuscript. HS and BN researched the data and reviewed/edited the manuscript. AP and FM contributed to discussion and wrote the manuscript. AM and CM contributed to discussion and reviewed/edited the manuscript. JB conceived of the study and reviewed/edited the manuscript. TB conceived of the study, researched the data, and wrote the manuscript.

## Conflict of Interest Statement

The authors declare that the research was conducted in the absence of any commercial or financial relationships that could be construed as a potential conflict of interest.

## Supplementary Material

Figure S1 Comparable regulatory T-cell (Treg) avatar suppression of polyclonal stimulated responder T cells (Tresp). **(A)** Tregs were transduced with GAD-reactive TCR clones (R164 or 4.13) or remained untransduced (Mock) and cultured with autologous polyclonal Tresp cells in decreasing proportions for 4 days with soluble anti-CD3 (2 µg/mL) and anti-CD28 (1 µg/mL) stimulation. Tresp proliferation was assessed by ^3^H-thymidine incorporation (left), and the percent suppression was determined by upon Tresp division index relative to the proliferation of Tresp when no Tregs were present (right). **(B)** IFN-γ production by polyclonal T cells was inhibited by Treg cells with or without TCR transduction. The levels of IFN-γ were measured from the supernatant by ELISA.
