# Supplementary material for: Avidity and Bystander Suppressive Capacity of Human Regulatory T Cells Expressing De Novo Autoreactive T-Cell Receptors in Type 1 Diabetes
Source: Front Immunol. 2017 Oct 26;8:1313. doi: 10.3389/fimmu.2017.01313 (PMC5662552; doi:10.3389/fimmu.2017.01313)
Supplement: Supplementary file 1 [file Data_Sheet_1.docx]

**SUPPLEMENTAL TABLES**

**Table S1. Experimental conditions to test polyclonal, antigen-specific, and bystander suppression of responder T cell (Tresp) activation by T cell receptor (TCR) transgenic regulatory T cells (Tregs) as shown in Figures S1 and 4.** Tregs (Row 2) were un-transduced (Mock), or transduced with lentivirus expressing the GAD-reactive 4.13 or R164 TCR clone as described in the methods section. Tresp (Row 3) were derived from peripheral blood mononuclear cells (PBMC), CD4^+^ conventional T cells expressing the 4.13 TCR (4.13 CD4^+^), or CD8^+^ T cells expressing the MART-1 TCR specific for Melan-A_27-35_ (MART-1 CD8^+^). Tresp were activated with irradiated antigen presenting cells (APC, Row 4) derived from HLA DRB*0401/A*0201 whole PBMC or CD3-depleted PBMC along with polyclonal stimulation (soluble anti-CD3/28 antibodies) or stimulation with antigen peptide(s), GAD557_555-567_ and/or Melan-A_27-35_ (Row 5).

|  | **Polyclonal Activation** | | | **Antigen-Specific Activation** | **Bystander Treg Activation** | **Bystander**  **No Treg Activation** |
| --- | --- | --- | --- | --- | --- | --- |
| **Treg** | Mock | 4.13 | R164 | 4.13 | 4.13 | 4.13 |
| **Tresp** | PBMC | PBMC | PBMC | 4.13 CD4^+^ | MART-1 CD8^+^ | MART-1 CD8^+^ |
| **APC** | PBMC | PBMC | PBMC | CD3^-^ PBMC | CD3^-^ PBMC | CD3^-^ PBMC |
| **Activation** | sCD3/28 | sCD3/28 | sCD3/28 | GAD_555-567_ | GAD_555-567_ & Melan-A_27-35_ | Melan-A_27-35_ |
