# Supplementary figures and images for: Avidity and Bystander Suppressive Capacity of Human Regulatory T Cells Expressing De Novo Autoreactive T-Cell Receptors in Type 1 Diabetes
Source: Front Immunol. 2017 Oct 26;8:1313. doi: 10.3389/fimmu.2017.01313 (PMC5662552; doi:10.3389/fimmu.2017.01313)

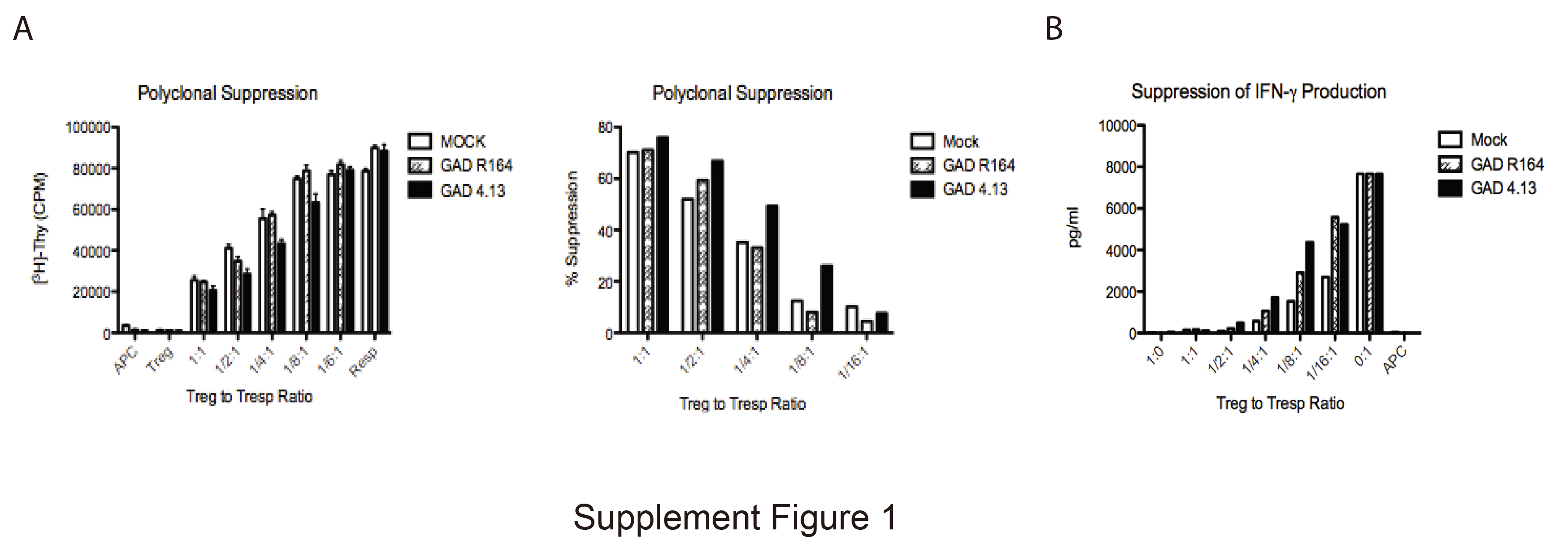

Supplement: Figure S1 — Comparable regulatory T-cell (Treg) avatar suppression of polyclonal stimulated responder T cells (Tresp). (A) Tregs were transduced with GAD-reactive TCR clones (R164 or 4.13) or remained untransduced (Mock) and cultured with autologous polyclonal Tresp cells in decreasing proportions for 4 days with soluble anti-CD3 (2 µg/mL) and anti-CD28 (1 µg/mL) stimulation. Tresp proliferation was assessed by 3H-thymidine incorporation (left), and the percent suppression was determined by upon Tresp division index relative to the proliferation of Tresp when no Tregs were present (right). (B) IFN-γ production by polyclonal T cells was inhibited by Treg cells with or without TCR transduction. The levels of IFN-γ were measured from the supernatant by ELISA. [file Image_1.jpeg]
